# Bilateral Vocal Cord Paralysis Following a Fall: A Rare Case With a Fatal Outcome

**DOI:** 10.7759/cureus.78895

**Published:** 2025-02-12

**Authors:** Wei-Chih Chen, Zi-Jie Lin, Kuan-Ting Lu

**Affiliations:** 1 Department of Neurosurgery, Taipei Veterans General Hospital, Taipei, TWN; 2 Department of Neurosurgery, Shin Kong Wu Ho-Su Memorial Hospital, Taipei, TWN

**Keywords:** airway intubation, bilateral vocal cord paralysis, bone spur, brain trauma injury, dysphonia, hyperextension of the neck, respiratory distress

## Abstract

Bilateral vocal cord paralysis is characterized by the immobility of the vocal cord and can lead to acute respiratory distress, requiring urgent airway intervention. This article describes a rare case of bilateral vocal cord paralysis following a fall. An 87-year-old female developed dysphonia after experiencing a fall-related accident. A brain computed tomography (CT) scan revealed a 4-mm-thick left tentorium subdural hematoma, without any bone or cartilage fractures. Cervical CT demonstrated degenerative changes from C4 to C7. Fiberscope examination confirmed bilateral vocal cord paralysis and atrophy. Hours later, she experienced stridor and hypercapnia, necessitating successful intubation. Vocal cord paralysis is rarely associated with blunt head injury; however, cervical hyperextension injuries and cervical spur compression have also been reported. The two factors were also highly suspected in our case, and they may be the causes of bilateral vocal cord paralysis. To conclude, the onset of dysphonia after trauma injury should raise concern for bilateral vocal cord paralysis and prompt consideration of airway protection.

## Introduction

The vocal cords are wedged-shaped structures and attached to the arytenoid cartilages. The movements of vocal cords contribute to vital functions of the larynx: breathing, swallowing, and speaking. Vocal cord paralysis is the loss movement of the vocal cord and is related to processes affecting the neurological control of vocal fold movement, including neuropathies of the vagus nerve or recurrent laryngeal nerve, neuromyopathies, or central neurologic issues. Studies indicate that iatrogenic injury, particularly from anterior neck surgeries, is the primary cause, followed by neoplasms and central nervous system lesions. Bilateral vocal cord paralysis accounts for 10% of all cases of vocal cord paralysis. Trauma has been reported to be present in 3% to 6.2% of bilateral vocal cord paralysis cases and 1.5% of all vocal cord paralysis cases [1,2].

The presenting complaints of patients with bilateral vocal cord paralysis include voice changes (such as hoarseness or breathing voice) and breathing difficulties (including stridor, dyspnea, and a risk of aspiration). A comprehensive history regarding the onset and duration of symptoms should be obtained, along with information on whether the symptoms are progressing or stable. Diagnosis is primarily made through clinical evaluation using flexible fiberoptic laryngoscopy, which allows for direct observation of the immobile vocal cords and assessment of their position [3]. We report a case of a patient who experienced immediate dysphonia and subsequently developed progressive respiratory distress and inspiratory stridor after suffering a fall episode.

## Case presentation

**Figure 1:**
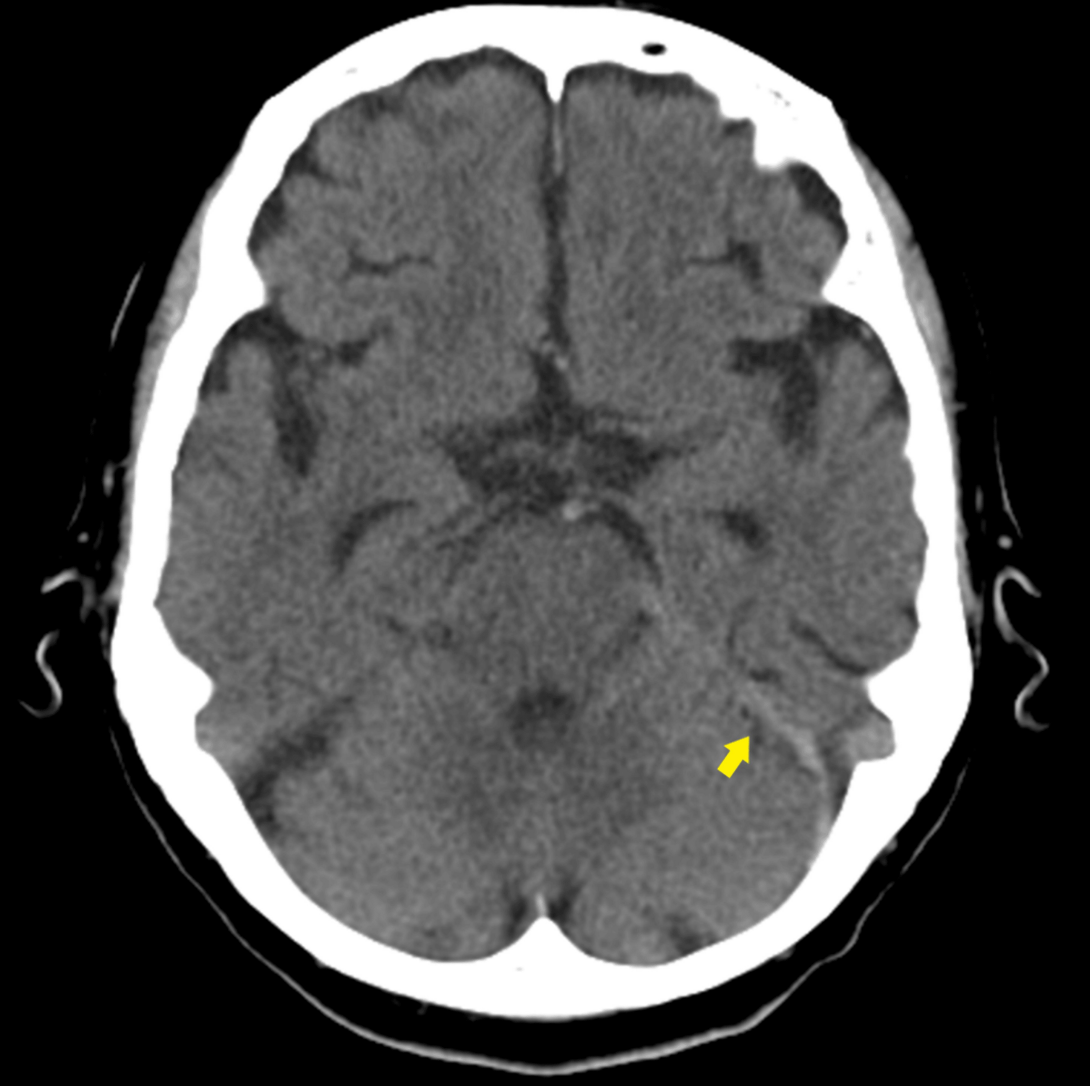
Brain CT A brain CT scan revealed a 4-mm-thick subdural hematoma along the left tentorium (yellow arrow), without midline shift or skull fracture.

**Figure 2:**
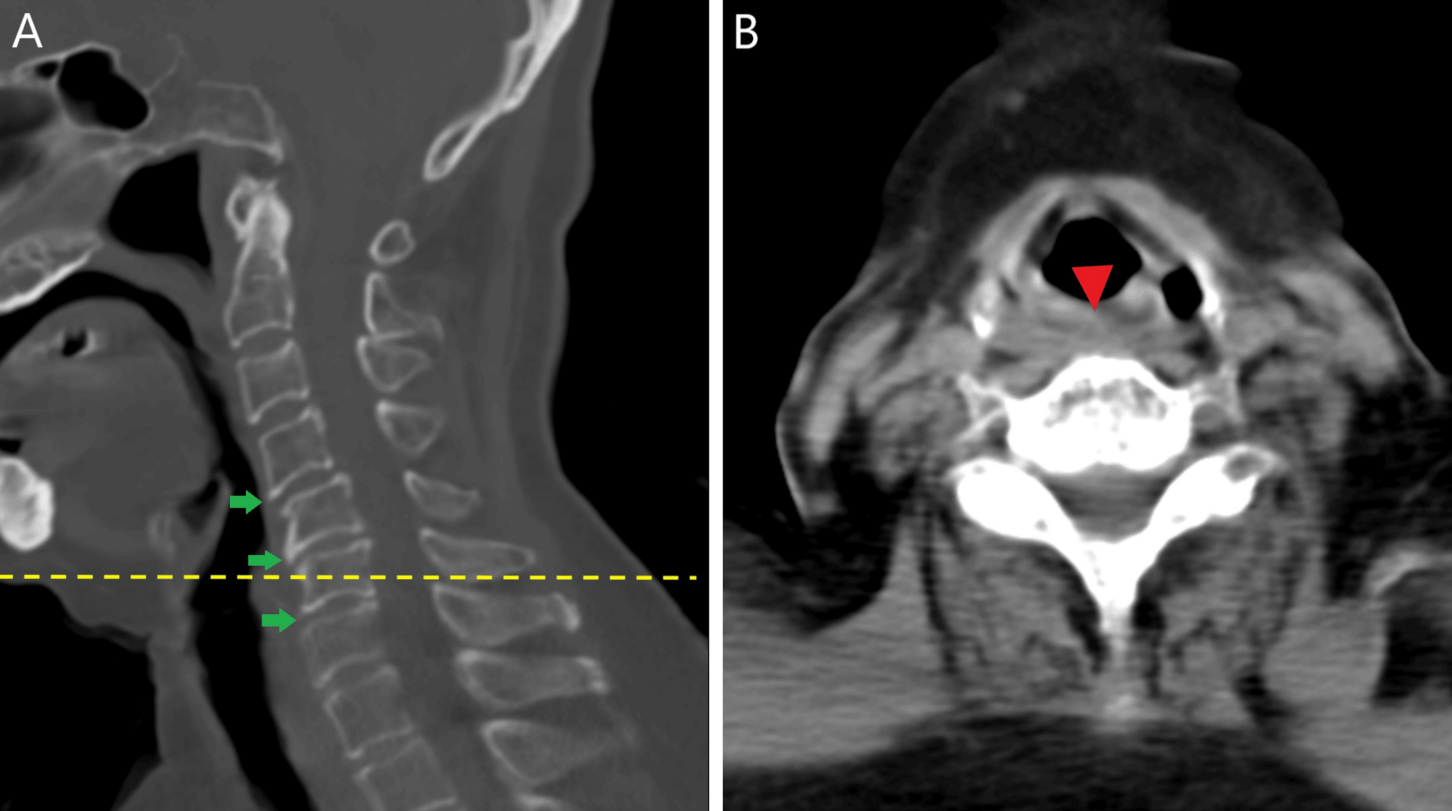
Cervical CT Cervical CT sagittal view (A) demonstrates mild spondylosis, disc space narrowing, and some spurs at levels C4–C7 (green arrow). The axial image (B) at C6 level (yellow dotted line) shows soft tissue swelling (red arrowhead), with no hypopharyngeal lesion and no evidence of cervical vertebral fracture no cartilaginous damage.

**Figure 3:**
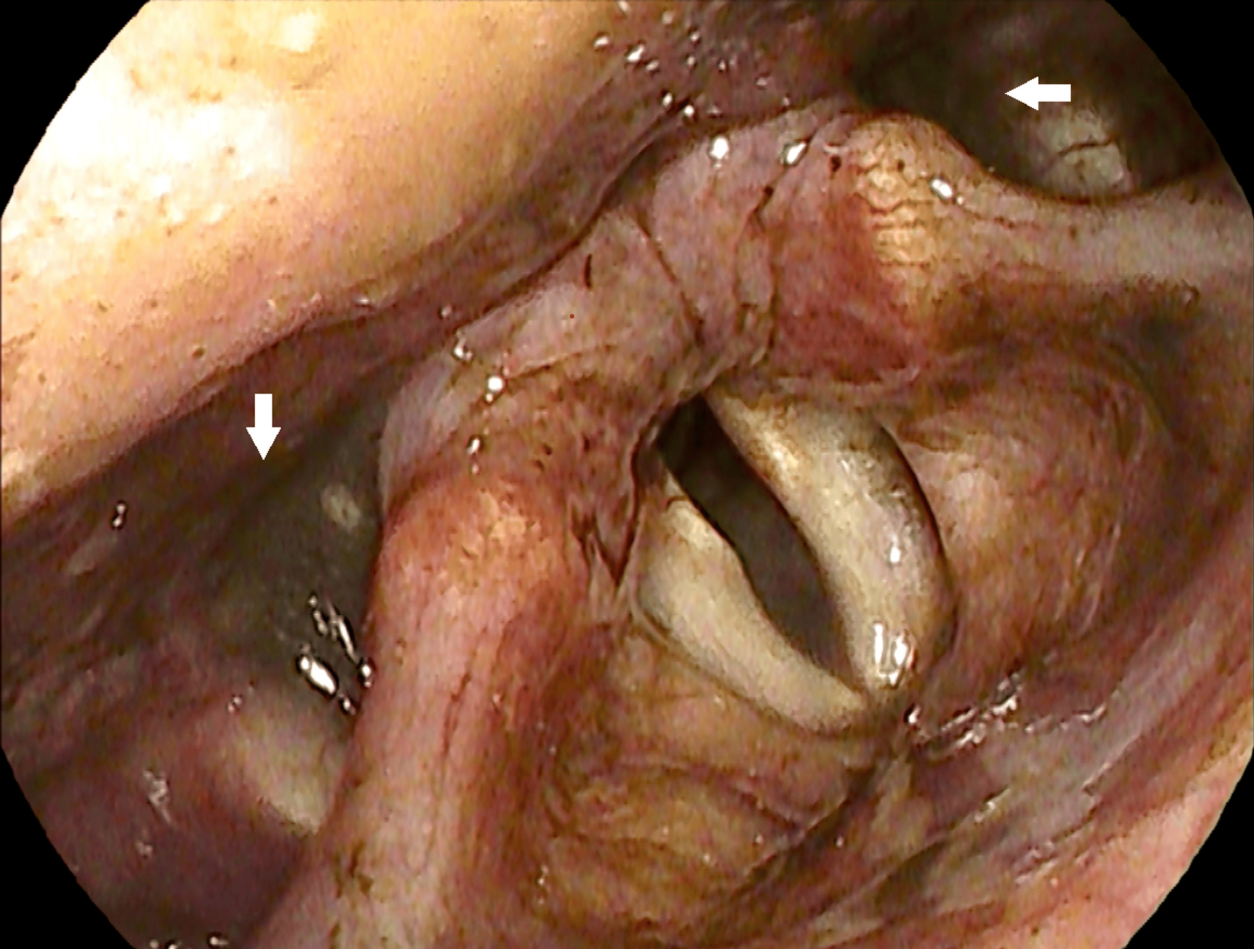
Fiberoptic laryngoscopy Fiberoptic examination revealed bilateral vocal cord immobility and atrophy in the paramedian position, accompanied by the pooling of dark-colored saliva (white arrow) in the hypopharynx. Glottic closure during phonation was severely incomplete. No edema, erythema, or masses were observed. Additionally, poor swallowing function was noted during the examination.

An 87-year-old female with a medical history of diabetes mellitus, dyslipidemia, and left breast carcinoma (status post-modified radical mastectomy 20 years ago with no recurrence) fell to the ground and suffered a contusion on her left forehead while hiking. According to paramedic reports, she developed dysphonia with a hoarse, low-pitched, breathy, and unclear voice immediately after the trauma, accompanied by headache, neck pain, and mild dizziness. There was no loss of consciousness or focal weakness, and she was promptly brought to the emergency department. On arrival, her vital signs were recorded as follows: blood pressure 130/94 mmHg, pulse rate 124 beats/minute, respiratory rate 16 breaths/minute, oxygen saturation 94% on room air, and body temperature 36.3℃. There was a 3-cm area of ecchymosis over the left forehead. The cervical spine was nontender and without deformity. The trachea was midline, and the oral cavity was clear. The Glasgow Coma Scale score was E4V2M6 (due to her breathy, unclear voice as incomprehensible sounds). Neurological examination revealed equal and reactive pupils, intact extraocular movements, symmetrical facial movements, and a preserved gag reflex; vagus nerve dysfunction was considered due to her hoarse and breathy voice. Muscle strength in all four limbs was intact, with full range of motion. Laboratory evaluations, including complete blood count and metabolic panel, were unremarkable. A chest radiograph showed no signs of acute cardiopulmonary disease. However, a brain CT scan revealed a 4-mm-thick left tentorium subdural hematoma (Figure 1). Cervical CT demonstrated no fracture but showed spondylosis and degenerative changes from C4 to C7 (Figure 2). Fiberoptic examination revealed bilateral vocal cord paralysis with atrophy, along with the pooling of dark-colored saliva in the hypopharynx (Figure 3).

Considering the high risk of choking and respiratory distress, otolaryngologists recommended prophylactic endotracheal intubation or tracheostomy for airway protection. However, the patient and her family declined these suggestions, thinking them to be too invasive and distressing. Several hours later at midnight, she developed stridor and hypercapnia, followed by bradycardia and cardiac arrest. After cardiopulmonary resuscitation and successful intubation, she regained spontaneous circulation but remained in a coma. A repeat brain CT showed no progression of the hemorrhage; however, hypoxic encephalopathy was suspected. Following thorough discussion with her family, the patient was compassionately extubated two weeks later and passed away shortly thereafter.

## Discussion

Producing the voice (phonation) and protecting the lower airways by maintaining glottic competence are the two primary functions of the vocal cords. The presentation and symptoms vary based on the underlying cause of the bilateral paralysis and the position of the vocal cords. Stridor and respiratory distress occur when the vocal cords are paralyzed in a more median position, although the voice may be normal, and there is minimal risk of aspiration. Conversely, the airway remains widely open but cannot effectively close when the vocal cords are paralyzed in a more lateral position. This typically results in significant voice issues, such as breathy voice, and an increased risk of choking or aspiration pneumonia, while complaints of stridor or respiratory distress are less common [3].

Bilateral vocal cord paralysis can result from vagus nerve injury anywhere along its pathway, from its origin in the medulla oblongata to its peripheral branches. The motor fibers of the vagus nerve provide innervation to the muscles of the soft palate, larynx, and pharynx via nucleus ambiguus. After leaving the skull through the jugular foramen, the vagus nerve descends into the neck area, initially accompanies the internal jugular vein medially in the carotid sheath, and lies between the carotid artery and internal jugular vein. Along its course, the vagus nerve divides into the superior laryngeal nerve, which further branches into an internal branch that supplies sensory innervation to the pharyngeal and laryngeal mucosa above the true vocal fold, and an external branch that innervates the cricothyroid muscle. The recurrent laryngeal nerve loops around the right subclavian artery on the right side and the aortic arch on the left side. It then ascends through the tracheoesophageal groove and enters the larynx posterior to the cricothyroid joint, approximately at the C6-C7 spinal levels [4]. Injury to the recurrent laryngeal nerve is the most common cause of vocal cord paralysis, typically resulting in a median position when bilateral or a paramedian position when unilateral. Injury to the superior laryngeal nerve can result in a lower pitch of the voice and may cause a bowing deformity of the vocal cords due to reduced tensile tone from the denervated cricothyroid muscles. A high injury to the vagus nerve may cause the vocal cords to remain in an abducted position, often described as "cadaveric" due to their widely separated appearance. This condition is frequently accompanied by other complications such as swallowing difficulties [3-5].

Cervical trauma resulting in bilateral vocal cord palsy has been previously reported. In a series of 1,182 cases of bilateral vocal cord paralysis in 2024, 3% (36/1182) of bilateral vocal cord paralysis were attributed to trauma [2,6]. Earlier studies of traumatic bilateral vocal cord paralysis often identified concurrent injuries, such as fractures of the thyroid cartilage or hyoid bone, retropharyngeal hematoma, laryngeal edema, skull fractures involving bilateral jugular foramen, or cervical spinal injury [7-11]. Neck whiplash or cervical hyperextension injuries have also been reported to cause bilateral vocal cord paralysis. Some authors hypothesize that cervical hyperextension injury creates traction on cranial nerve fibers including the vagus nerve and the vertebrobasilar circulation supplying them. The recurrent laryngeal nerve, which innervates the vocal cords, may be particularly susceptible to blunt trauma near the thyroid cartilage due to its superficial course [12,13]. Moreover, in the tracheoesophageal groove, a pre-existing bone spur can compress and damage the nerve, leading to vocal cord paralysis [14]. In our case, the mechanism of falling down with forehead contusion injury suggests a neck hyperextension injury, even though no structural damage to the cervical spine was identified. Some C6/7 spurs were also observed on the cervical CT, which may indicate compression of the tracheoesophageal groove. In patients with vocal cord paralysis related to trauma, management should include physical examinations assessing neck and airway appearance, respiratory conditions, other cranial nerves, and muscle strength in all four limbs. Cervical imagings, such as X-rays and CT scans, will be required to evaluate whether there is a combination of a cervical injury, cartilage fracture, hyoid bone fracture, hematoma, or spur compression. Furthermore, a fiberoptic examination of the vocal cords and consideration of prophylactic airway protection are recommended to prevent potential complications.

## Conclusions

Bilateral vocal cord paralysis can lead to various degrees of dyspnea and may be life-threatening. We report a case of bilateral vocal cord paralysis following a fall. Neck hyperextension caused by the head injury along with cervical spur compressed to the tracheoesophageal groove were highly suspected to have resulted in recurrent laryngeal nerve palsy. This case supports previous reports indicating that blunt head trauma and neck hyperextension can damage the recurrent laryngeal nerves, causing vocal cord paralysis. Clinicians should be aware that dysphonia following blunt trauma may indicate an airway emergency.
